# Binocular Information Improves the Reliability and Consistency of Pictorial Relief

**DOI:** 10.3390/vision7010001

**Published:** 2022-12-22

**Authors:** Paul B. Hibbard, Rebecca L. Hornsey, Jordi M. Asher

**Affiliations:** Department of Psychology, University of Essex, Colchester CO4 3SQ, UK

**Keywords:** binocular vision, depth perception, 3D vision, stereopsis, binocular disparity, pictorial relief, natural images, depth-cue combination, virtual reality

## Abstract

Binocular disparity is an important cue to three-dimensional shape. We assessed the contribution of this cue to the reliability and consistency of depth in stereoscopic photographs of natural scenes. Observers viewed photographs of cluttered scenes while adjusting a gauge figure to indicate the apparent three-dimensional orientation of the surfaces of objects. The gauge figure was positioned on the surfaces of objects at multiple points in the scene, and settings were made under monocular and binocular, stereoscopic viewing. Settings were used to create a depth relief map, indicating the apparent three-dimensional structure of the scene. We found that binocular cues increased the magnitude of apparent depth, the reliability of settings across repeated measures, and the consistency of perceived depth across participants. These results show that binocular cues make an important contribution to the precise and accurate perception of depth in natural scenes that contain multiple pictorial cues.

## 1. Introduction

Binocular vision provides us with precise information about depth, in principle allowing us to determine the exact three-dimensional location and shape of objects within our environment. As a result, binocular cues contribute to many everyday tasks, including navigating around our environment [1,2], reaching out to pick up objects [3,4,5,6,7], and recognising three-dimensional shape [8].

In a typical everyday scene, binocular disparity is but one of many cues to depth, along with occlusion, perspective, shading and motion, amongst others [9]. Each of these cues provides useful but uncertain information about the 3D properties of the environment [10,11]. Our visual system is able to combine these cues so as to reduce uncertainty [11,12,13,14,15,16]. Statistical models of this process have been developed, based on the idea that uncertainty can be minimised if depth cues are weighted in proportion to their reliability [11,12,17,18]. Empirical results have shown that the combination of depth cues can be close to optimal [12,13,14,16,19,20].

A major limitation in our understanding of this process is that we have not quantified the contributions that different cues make to the perception of depth in everyday vision. In a typical investigation of how depth cues are combined, the experimenters will carefully isolate, quantify, and manipulate two or more cues, in order to determine how each contributes to the perception of depth [14,15,16]. This then allows them to judge the extent to which the presence of multiple cues improves the precision of depth estimates, and whether cues are weighted in an optimal fashion that takes account of their relative reliability. It is this careful isolation of cues, in highly artificial situations, that has allowed researchers to precisely quantify the degree to which human vision conforms to the optimal statistical solutions in some cases [12,13,14,16,21] but not in others [22,23,24].

It is not however possible to predict from these studies the importance of binocular disparity or other cues in everyday vision, since this will depend on the particular balance of information provided by the many depth cues available in any given scene [25]. To answer this, it is necessary to design experiments using complex natural stimuli [26,27]. However, the use of complex stimuli in this context is relatively uncommon [28,29], and the use of naturalistic stimuli even more so. As a result, we have surprisingly little understanding of how much, and in what way, binocular disparity contributes to the perception of depth in everyday life. The focus of the current study is to assess the contribution of binocular vision to depth judgements in photographs of complex natural scenes.

Photographs of real objects have been used as stimuli in the perception of pictorial relief [30,31]. Static photographs can contain all of the pictorial cues available in the natural environment, and as such create a strong and convincing sense of three-dimensional space. The structure of this perceptual experience can be explored using a range of depth probes, designed for example to allow users to report the depth order of points, the local three-dimensional orientation of surfaces, or the cross-sectional shape of objects [32,33,34]. Studies using these techniques have shown that it is possible to reconstruct a depth map, indicating that these local settings are consistent with a global surface structure [30,32,33].

This approach provides a number of important benefits. First, the use of natural images as stimuli ensures a clear phenomenal experience of three-dimensional structure. Second, photographs contain a wealth of pictorial cues, and importantly these cues are physically created, rather than being rendered artificially with potential artefacts [35]. Third, it is possible to manipulate individual aspects of the stimuli parametrically, such as lighting and viewing directions, while ensuring that all other pictorial cues are available [30,36,37].

The perception of depth relief in photographs is distinct from the perception of depth structure in the three-dimensional environment. The structure of pictorial relief exists within the picture, which is itself perceived as an object in three-dimensional space in its own right. It also differs in terms of the depth cues that are available, and in how these interact with the viewer. In a photograph, the parallax cues of motion, accommodation, binocular disparity, and vergence do not provide information about the structure of the scene. Moreover, when the observer moves, the retinal image changes in a way that is consistent with the picture surface, rather than the scene within the picture. These properties are an important distinction between the perception of pictorial relief, and the perception of depth in three-dimensional space, or in virtual reality.

The perception of pictorial relief defines not just the apparent three-dimensional structure of surfaces in the scene, but also the observer’s location relative to these surfaces. Our perception of space is that of seeing the world from a particular point of view [38,39]. This is the location from which, at any one point in time, we are sampling the ambient optic array [40]. When viewing a picture, this will be a point in pictorial space, rather than the viewer’s location in their own environment. In this case, it might be expected that this would correspond to the location of the camera in the scene. However, it has been found that there is considerable variability in the gauge settings made by different observers, and that this variation can be characterised as a change in the location from which the scene is observed [34]. There is thus a “beholder’s share”, the contribution that the individual brings to the interpretation of the image [41] that consists of the point of view from which pictorial space is experienced.

An important difference between monocular and binocular viewing in this regard is that binocular information about the viewing geometry allows us to unambiguously specify the 3D locations of points relative to the observer [42,43,44]. This reduction in uncertainty means that changes in the position from which images are viewed have a greater effect on apparent depth under stereoscopic viewing [45,46,47,48]. As a result, the problem of viewing pictures from the incorrect vantage point is exacerbated by the presence of stereoscopic depth cues. This is consistent with the role played by these cues in providing less ambiguous information about metric depth, and providing an experience of depth as perceived from a well-specified point of view. In previous studies of stereoscopic viewing, stimuli have been relatively impoverished, such that other pictorial depth cues may have played a relatively small role in determining the vantage point. This is because differences in the effects of an incorrect viewing position between stereoscopic and non-stereoscopic viewing diminish as the amount of pictorial depth information is increased [48]. Conversely, when the observer’s location coincides with the camera positions from which the photographs were taken, stereoscopic cues should provide accurate and precise information about the viewer’s position in the scene.

Stereoscopic photographs thus represent an intermediate case between single photographs and the three-dimensional space of the real or virtual world. In the case of orthostereoscopic capture and display, the cameras are positioned, and the photographs displayed, so that the images are the same as would be experienced by the observer viewing the scene from that location [49]. Under stereoscopic viewing, retinal and extra-retinal cues provide information about the location of this point of view relative to the scene [42,43,44]. This contrasts with standard picture viewing, in which the same retinal- and extra-retinal binocular cues provide information about the observer’s location relative to the picture surface, and the location and orientation of that surface.

Orthostereoscopic viewing is not however typical of our experience with stereoscopic photographs and movies [48]. This is because both the capture and presentation of the image, for every frame, need to be carried out in such a way that they replicates the optic array that would be experienced in the real environment. The positions of the cameras determine the two locations from which the ambient optic array is sampled. A requirement for orthostereoscopic viewing is that the intercamera separation needs to be to tailored to the viewer’s interpupillary distance. The orientations of the cameras then determine which regions of the optic array are sampled. The camera geometry, including the location and orientation of the imaging plane, specifies how this optic array is projected to create the resulting image. Since there is a one-to-one mapping between visual directions in the optic array and locations in the image, these factors do not affect the information that is captured in the image. However, the optic array experienced by the observer viewing an image on a display screen is influenced by both the camera and display screen geometry, and appropriate transformations between the two are required in order to create an orthostereoscopic view. While it is possible through this approach to ensure the appropriate optic array, focus cues will typically be inconsistent with this optic array. In particular, while in the natural environment observers will accommodate and converge to the same distance, this is only true in a stereoscopic display for points that have zero disparity on the display screen.

While orthostereoscpic viewing is typically approximated in virtual reality, there are multiple factors that in practice contribute to deviations from orthostereoscopy in picture viewing. These include the separation and orientation of the cameras [50,51], the transformation of the image between the camera and display screen [52,53,54], and the location of the observer relative to the screen while viewing [48,55].

When viewing is not orthostereoscopic, there will be a mismatch between the ground truth three-dimensional structure of the scene, and the structure specified by the binocular retinal images. Despite this mismatch, observers are able to use binocular information to estimate their location relative to the scene and in turn use this to estimate the three-dimensional structure of the viewed objects [43,56,57,58]. In many cases of photographic rather than computer-rendered stimuli, the ground truth, and thus the mismatch between the physical and experienced space, will be unknown. However, stereoscopic viewing will still provide precise information about metric depth even in non-orthostereoscopic viewing conditions.

Given the precise and consistent information that is available under stereoscopic viewing, we can predict two differences in the perception of pictorial relief in comparison with standard picture perception. The first is that gauge figure settings will be more precise and reliable. The second is that the beholder’s share will be reduced, and that settings will become more consistent between observers.

Previous studies of the effects of binocular cues on the perception of pictorial relief have considered cases where these cues provide information about the surface of the picture (in single photographs) and cases where they provide information about the scene within the picture (in stereoscopic photographs). In the former case, binocular cues are expected to diminish the perception of pictorial relief, while in the latter they are expected to enhance it.

Binocular cues provide information about the flatness of the picture surface in standard binocular viewing of a photograph, and in this case gauge settings have been described as a compromise between the orientation of the picture surface, and the orientation of surfaces seen within the picture [59]. Binocular information about the picture surface can be removed by monocular or synoptic viewing. In the latter case, the same image is presented to each eye with parallel vergence, creating zero disparities across the image. Greater depth relief is perceived in both of these conditions, consistent with the removal of the influence of binocular cues to the flatness of the picture surface itself [59].

The contribution of binocular disparities that signal the structure of the depicted scene, rather than the picture plane, has also been studied [37]. In this case, photographs were taken with a stereoscopic camera with a stereo baseline of 0 cm, 7 cm (close to the adult average of 6.3 cm [60,61]), and 14 cm. Increasing the stereo baseline increases the size of the binocular disparities, and should lead to an increase in perceived depth. Such an increase was found, although it was much reduced compared to the effect predicted if observers had based their judgements only on disparity. This is consistent with a substantial contribution of pictorial cues to depth relief. In this experiment, stimuli were always presented binocularly. This means that, in the 0 cm baseline case, where binocular cues provided no information about the structure of the scene, they instead indicated the location and orientation of the picture itself.

Monocular and binocular viewing of similar photographs was also compared in a different study [36]. Observers made the same local surface orientation judgements for objects in photographs viewed monoculary, or binocularly in image pairs taken with a stereoscopic camera with a baseline of 7 cm. Perceived depth tended to be greater in the binocular condition. While this indicates the contribution of binocular cues to apparent depth, no difference between conditions would be predicted if pictorial and binocular cues were providing consistent depth information. This would only, however, be expected if the viewing geometry of both image capture and display were matched to each individual participant to provide accurate binocular cues. The decreased uncertainty about the point of view with binocular cues might also be expected to increase the consistency of responses between participants. However, correlations between settings for pairs of participants did not differ between monocular and binocular viewing.

The aim of the current study was to assess the contribution of binocular cues to the reliability and consistency of the perception of pictorial relief in photographs of natural scenes. Cue combination models predict that the combination of multiple cues will lead to more precise estimates of depth [11,12,13,17,18]. This means that depth judgements would be more reliable, and more similar over repeated measures. While this has been shown in many studies using simple, artificial stimuli [12,13,14,15,16,20,21] in which the relative reliability of cues is carefully controlled, here we tested whether precision is increased in complex natural scenes, and if so by how much. Additionally, we assessed whether the presence of binocular cues affects the amount of depth relief perceived. If all depth cues are unbiased, as is typically assumed, then the addition of binocular cues should increase the magnitude of depth perceived, due to the reduced influence of residual focus cues to the flatness of the display screen [62]. However, it is known that the perception of depth from individual cues is often biased [63,64] and that in these cases cues are nevertheless combined in a way that increases the precision of depth estimates [19,20]. Given these potential biases, and that we did not use orthostereoscopic viewing, combining binocular and pictorial cues might alter the perceived depth relief. In particular, we used cameras with a fixed separation that were converged on a target object, and presented the images as binocular photographs, rather than attempting to recreate the ambient array from the cameras’ locations for the viewer. This ensured that there was no conflict between accommodation and convergence for the camera fixation point. Thus, we do not make any specific predictions about how the magnitude of perceived depth is affected by the presence of binocular cues.

We also assessed the effect of binocular cues on the consistency of pictorial relief between observers. These variations have been described as a change in the viewing position between observers [34]. The increased information about this viewing location available in stereoscopic images means that this variability is predicted to decrease.

## 2. Materials and Methods

### 2.1. Apparatus

Stimuli were presented on a VIEWPIXX 3D monitor (VPixx Technologies, Saint-Bruno, QC, Canada), viewed from a distance of 96 cm. The monitor screen was 52 cm wide and 29 cm tall. The screen resolution was 1920 × 1080 pixels, with a refresh rate of 120 Hz. Each pixel subtended 1 arc min. Stimuli were presented at 8-bit resolution. Stereoscopic presentation was achieved using a 3DPixx IR emitter and NVIDIA 3D Vision LCD shutter glasses (NVIDIA, Santa Clara, CA, USA). Gauge settings were made using the computer mouse. Stimuli were generated and presented using MATLAB (Mathworks, Natick, MA, USA)and the Psychophysics Toolbox extensions [65,66,67].

### 2.2. Participants

Eight participants (4 male, 4 female) took part in the experiment, including the experimenters RH and PH. Interpupillary distance (IPD) was not measured, and the same stereoscopic photographs were presented to all observers.

### 2.3. Stimuli

**Figure 1:**
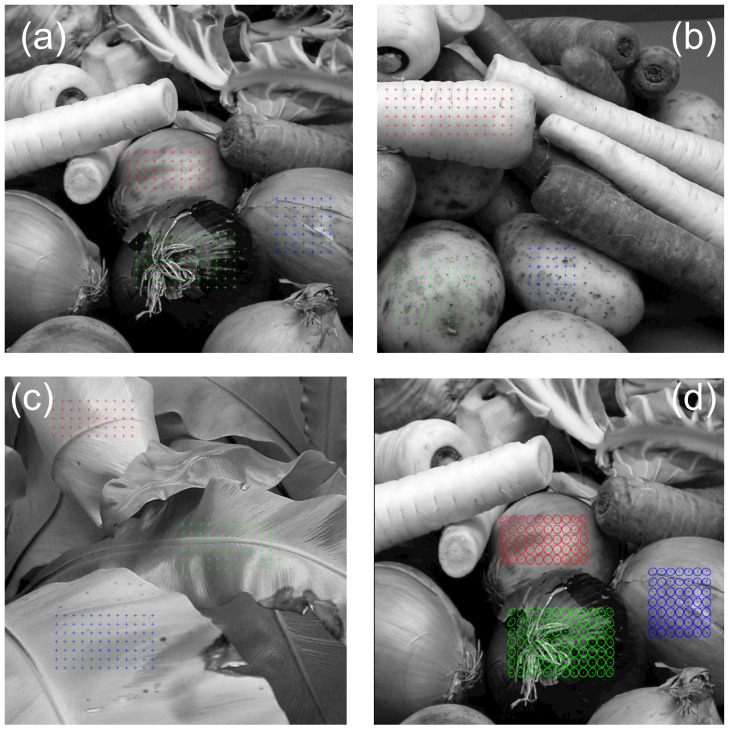
(**a**–**c**) The three scenes used, with the crosses marking the sampled locations for each of the three settings; (**d**) average gauge settings for one scene.

Stimuli consisted of three greyscale stereoscopic photographs of vegetables and plants (Figure 1a–c). The methods used to capture these photographs are detailed elsewhere [68] and summarised here. The images were captured using two Nikon Coolpix 4500 digital cameras, harnessed in a purpose-built mount with the inter-camera separation fixed at 6.5 cm (Nikon, Tokyo, Japan). This compares with an average interpupillary distance of 6.3 cm in human adults [60,61]. The cameras were oriented so that the same point in the scene projects to the centre of each camera’s image, and were transformed using a pin-hole camera model to take account of the relationship between visual direction and the camera screen. The resolution of the stimuli was 1 arc min per pixel; the images were 20 degrees square. In the psychophysical experiments, the images were presented with the same resolution. Given the variation between the capture and display geometry, and that the distance to the fixated point in the photographs is not known, our intention was not to produce orthostereoscopic viewing, but to assess the effect of the presence of binocular cues on the precision and reliability of the perception of pictorial relief.

The participants’ task was to indicate the apparent 3D orientation of the surface of the object present at a number of pre-specified locations in the scene. In each photograph, we selected three rectangular regions on three separate objects. A rectangular grid of positions, separated horizontally and vertically by 30 arc min, was defined within each region as the locations at which the surface orientation was to be estimated. The number of positions sampled varied between 36 and 90 between objects; a total of 595 points were probed in total across the three photographs.

Participants indicated the apparent 3D orientation of the surface using a gauge figure that consisted of an ellipse and a straight line (Figure 1d). The gauge figure was presented in red; the thickness of the line was 1 pixel. The length of the line when it was parallel to the screen was 10 pixels, and the diameter of the ellipse, when circular, was 20 pixels. The aspect ratio of the ellipse, and the orientation of the ellipse and line, were controlled by the observer using the mouse. Their task was to orient the gauge figure so that the ellipse appeared aligned with the orientation of the surface, and the line coincident with the surface normal.

### 2.4. Procedure

In each block of trials, the observer was presented with one of the three photographs. This was either presented stereoscopically, or monocularly to the observer’s dominant eye. In the monocular case, the image presented to the other eye was black. The gauge figure was in both cases presented monocularly, to the participant’s dominant eye. Monocular presentation ensured that the gauge figure did not contain any binocular cues to its location in depth.

On each trial, the gauge figure was presented at one of the pre-specified locations; its initial orientation was randomised. The participant manipulated the orientation of the gauge figure using the mouse, until it appeared aligned with the 3D orientation of the object at that location on the surface. When they were satisfied with the orientation they had set, they clicked the mouse button, and the gauge figure moved to a new location for the next trial. Participants completed three blocks of trials for each photograph, for both monocular and stereoscopic viewing. They thus completed a total of 18 blocks of trials (3 repetitions × 3 photographs × 2 viewing conditions).

In addition five participants completed an additional 9 repetitions for the first photograph to give a total of 12 repetitions for each point in this scene. These additional data were used to compare the variability in responses across trials between monocular and binocular viewing. The results presented here are thus based on more than 80,000 individual gauge settings.

## 3. Results

### 3.1. Analysis

**Figure 2:**
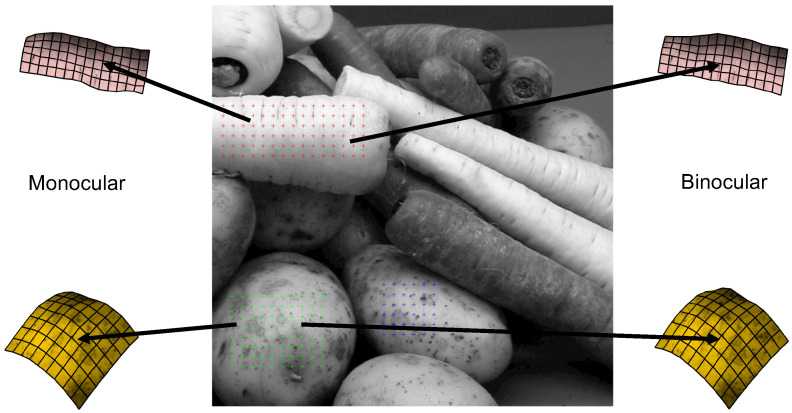
Example depth maps, averaged over participants, for two objects under monocular and binocular viewing.

For each trial, the slant and tilt of the gauge figure were recorded. From these values, we can generate a relief map—the depth surface that provides the best fit with the orientation settings made [32]. This procedure is outlined in detail by Nefs [69]. Relief maps were calculated from the mean of the settings made across the three repetitions. An example of the gauge figure settings is shown in Figure 1d, and the relief maps that were fit to these settings in Figure 2. In this relief map, depth is specified for each point on the sampling mesh relative to the distance in the image plane. We applied this technique to create a depth relief map for each of the nine objects (three for each scene) separately, for monocular and stereoscopic viewing conditions. These depth maps were used as the basis for assessing the effects of binocular viewing on depth magnitude, reliability, and consistency between participants.

### 3.2. Magnitude

We first assessed the effect of binocular cues on the magnitude of perceived depth. Each of the nine relief maps defines a depth location for each of the sampled points on that object. When combined, these provided a total of 595 samples. These depth maps describe the relative depth between the points on the surface. The minimum depth value was subtracted from each map, so that the furthest distance was in all cases 0 mm.

**Figure 3:**
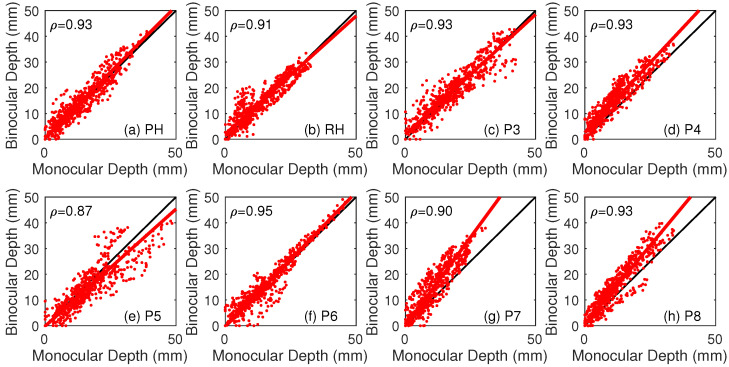
(**a**–**h**) Depth values for each point under monocular and binocular viewing for each participant. The black line shows a slope of 1, and the solid red line shows the slope of the total least squares regression. Pearson’s correlation coefficient ρ is given for each participant.

Scatter plots of the monocular and binocular depth values and the fitted regression lines are plotted in Figure 3. Correlation coefficients for depth perceived under the two viewing conditions varied between 0.87 and 0.95 between participants, with a mean of 0.92. These show a strongly linear relationship between the two conditions. We then calculated a regression analysis for each participant, to compare depth values between monocular and binocular viewing. A difference in the amount of depth between viewing conditions would lead to a regression slope that differed from 1. Since both of these measures are dependent variables, total least squares was used, so that the residual errors were minimised in the direction orthogonal to the estimated regression line [70]. Slopes of these functions varied between 0.91 and 1.38, with a mean of 1.06. A single sample *t*-test showed that the mean slope did not differ significantly from 1 (t(7) = 1.11, *p* = 0.30, Cohen’s D = 0.39). For each of the scene locations, the average depth was also calculated across participants, for each viewing condition. A pairwise *t*-test showed that the mean depth was smaller under monocular (mean = 14.5 mm, SD = 78 mm) than binocular (mean = 15.98, SD = 8.26 mm) viewing (t(594) = 24.80, *p* < 0.001, Cohen’s D = 0.19). This comparison indicates an average increase in the magnitude of perceived depth of 10.5%.

### 3.3. Reliability

For the first scene, gauge settings were repeated 12 times for 5 of the participants. This allowed us to create a depth relief map for each replication and assess the reliability of depth relief by calculating the standard deviation for each point across repetitions. When the standard deviation was averaged across participants, reliability was significantly greater under binocular (mean = 1.54 mm, SD = 0.39 mm) than monocular (mean = 1.74, SD = 0.44 mm) viewing (t(179) = 7.16, *p* < 0.0001, Cohen’s D = 0.47).

### 3.4. Consistency

We quantified the consistency of depth settings between observers by comparing the depth values obtained across all pairs of participants, obtained separately under monocular and binocular viewing [34]. As we had eight participants, there were 28 different pairings of participants. For each pair, we calculated the root mean square (RMS) differences between the depth values, the correlation coefficient, and performed a total least squares regression on these data. The value of the slope obtained for this regression will depend on how the participants are assigned to the *x* and *y*-axes. Since this decision is arbitrary, data were assigned so as to ensure that the slope was greater than 1. This means that larger values indicate that a greater stretch in depth was required to map the data from one participant to another. Regressions were performed separately for the monocular and binocular conditions.

The RMS error was smaller under binocular (mean = 5.52 mm, SD = 1.12 mm) rather than monocular (mean = 6.42 mm, SD = 1.64 mm) viewing (t(27) = 3.57, *p* = 0.0014, Cohen’s D = 0.64). Perceived depth was thus more consistent between participants when binocular cues were available.

Correlation coefficients indicated a good linear relationship between pairs of participants. These varied between 0.67 (*p* < 0.001) and 0.93 (*p* < 0.001) for monocular viewing and 0.65 (*p* < 0.001) and 0.94 (*p* < 0.001) for binocular viewing. Correlations did not differ between monocular (mean = 0.82, SD = 0.073) and binocular (mean = 0.83, SD = 0.07) viewing (t(27) = −1.10, *p* = 0.28, Cohen’s D = −0.19).

Regression slopes were closer to 1 for binocular (mean = 1.15, SD = 0.117) than monocular (mean = 1.27, SD = 0.178) viewing, again indicating closer agreement between participants when binocular cues were available (t(27) = 2.84, *p* = 0.0084, Cohen’s D = 0.76).

## 4. Discussion

### 4.1. Magnitude

The range of pictorial depth was 10% greater under binocular viewing. There are a number of reasons why this difference might have occurred, which can be classified as (1) the influence of other depth cues, (2) biases in the depth specified by binocular cues, or in how this is perceived or (3) evidence for a process of cue-combination that deviates from weighted averaging.

In traditional picture viewing, some depth cues provide information about the picture surface, rather than the scene within the picture. These include motion parallax, binocular disparity and vergence, and focus cues. Any influence of these cues in the current study would tend to flatten the depth relief perceived [59]. This would tend to increase the depth perceived in the binocular case, since the introduction of a reliable depth cue would tend to decrease the weight attributed to other cues, including these picture flatness cues.

Our experiment was designed to reduce these flatness cues by comparing stereoscopic viewing with monocular viewing, thus removing binocular cues to the screen plane in the latter condition. Some flatness cues will have remained, however. Firstly, although the participants’ position was maintained using a chin-rest, any residual movement would have provided motion parallax information indicating the structure of the surface of the display screen. Second, when the participants made eye-movements around the scene, to locations at different depicted depths, these were not accompanied by the expected changes in accommodation and image blur [62,71,72,73]. These focus cues tend to conflict in traditional pictures and displays, but may be rendered in a way that is consistent with the scene content using a multi-focal display [74,75].

An alternative explanation is that the binocular information either specified, or was interpreted as, a greater depth range. Under orthostereoscopic viewing, in principle, the cues from both binocular and pictorial cues would be unbiased, and combining them would not affect perceived depth. However, this would require a perfect correspondence between the viewing geometry of the scene capture and display, and an unbiased interpretation by the participants. Our presentation of the photographs was not intended to achieve orthostereoscopic viewing, and there were a number of important ways in which our stimuli would have deviated from this. The first is that a fixed inter-camera distance of 6.5 cm was used, meaning that disparities could not be corrected to match each individual observer’s IPD. Since the average adult IPD is 6.3 cm [60,61], disparities are likely to have been larger than they would be under natural viewing, although not to the extent that they would account for the perceptual stretching in depth observed. The second difference is that the distance from the observer to the screen was fixed, whereas the distance to the fixated point in the centre of each photograph varied. This again would have introduced a discrepancy between the intended and perceived depth structure. The final possibility is that, even if binocular information was consistent with pictorial cues, there are known biases in the way that this information is perceived [20,63].

Finally, while the weighted averaging model predicts that the magnitude of perceived depth should not be affected by the number of cues available, other models predict that perceived depth should increase when binocular cues are added. For example, Tyler’s accelerated cue combination principle predicts that perceived depth will increase with the number of depth cues available, asymptoting to veridical perception under full cue conditions [76]. Our results are consistent with his observation that apparent depth is reduced under monocular viewing.

### 4.2. Reliability

The variability in depth maps over repeated settings was lower with binocular viewing. This increase in reliability is predicted from the increase in precision when binocular cues are available [11,14,15,77]. As the gauge settings require participants to indicate the perceived three-dimensional orientation of surfaces, these data show that the increase in precision of these judgements in simple stimuli [14] is also found in photographs of natural scenes.

### 4.3. Consistency

Previous studies have shown considerable variation in pictorial relief across different observers [34,36,37], and that these differences can be partly accounted for by a different effective viewpoint from which surface orientation judgements were made [34]. We found that depth relief was more consistent across observers when binocular depth cues were available. This is likely to reflect the fact that binocular cues, including vergence and gradients of horizontal and vertical disparity, can be used to specify the point of view precisely [42,43,44,78,79,80].

When viewing from an incorrect distance or direction, distortions in perceived shape are expected to be greater under stereoscopic viewing, due to the mismatch between the locations from which the images were captured and viewed [48,81,82,83]. Previous research has shown that our ability to compensate for viewing pictures from the wrong location is reduced under stereoscopic viewing [48,83,84,85]. Our results suggest that these distortions will also be more consistent between observers.

### 4.4. Veridicality and the Operational Definition of Pictorial Relief

Our pictorial depth relief measures provide a description of the relative depth of points on objects that is most consistent with the slant and tilt settings made by observers. These measures can be used to compare perceived depth between viewing conditions, observers, and repeated sessions. In this case, we found a greater depth range, reliability and consistency when binocular cues were available. These results are all consistent with a reduced influence of display screen flatness cues, and the greater precision and reduced ambiguity of pictorial depth information when binocular cues are available. No ground truth data regarding the physical structure of the objects in the photographs were available, meaning that comparisons between pictorial and physical space cannot be made. Experiments such as in the current study are restricted to comparisons between results within pictorial space, and cannot address the question of how veridical pictorial space is in comparison with physical space.

Another form of veridicality that can be considered, however, is the extent to which the slant and tilt settings made by observers accurately reflect the apparent orientation of surfaces in the scene. It has been argued that there will always be an unknown mapping between the property of perception to be reported on, and the way that it is reported [62], and in many cases it is simply assumed that perception is unbiased in comparison with physical space in order avoid this complication [64]. In contrast, it has also been argued that pictorial relief is operationally defined by the measurement procedure that has been adopted [30], and that the question of how observers’ settings relate to the publicly inaccessible perceptual experience outside of the measurement process is misplaced. It is then possible that different types of gauge figure will yield different results [34].

In the current study, we used the same gauge figure, presented in the same way, for our two viewing conditions. It remains a possibility that the perception of the gauge figure, rather than the photograph, was altered by the provision of binocular information. While this cannot be ruled out, the facts that the gauge figure itself was unaltered between conditions, and that the differences found are consistent with the predicted increase in precision of pictorial relief, make this the less parsimonious interpretation.

### 4.5. Binocular and Pictorial Information in Stereoscopic Photographs

Different depth cues provide different types of information that may influence different types of gauge figure settings in different ways. For example, occlusion can tell us the depth order of objects, texture gradients the orientation of surfaces, and parallax cues such as binocular disparity the full metric structure of the scene. In natural photographs, in comparison with simple laboratory stimuli, it is by no means trivial to specify the contributions of all cues to the perception of depth. However, the procedure adopted here, of varying one cue while keeping all others constant, allows us to assess its contribution at a natural operating point set by all other cues [30]. The contributions of other cues, such as surface texture and shading, could be addressed by independently varying them within the same scene. This is more readily achieved using computer-rendered stimuli rather than photographs of real scenes.

### 4.6. Implications for Virtual Reality and Everyday Vision

Our results suggest that binocular cues will increase the precision of depth perception in everyday, real-world vision, and that the provision of binocular cues in virtual reality will provide a similar increase in precision. In the current case, binocular cues increased the magnitude of perceived depth by 10.5%, reduced the standard deviation of depth relief across repeated setting by 11%, and reduced the differences between observers by 16%.

It is important to note that the lack of consistency in pictorial relief between observers that has been found [34] relates to the perception of pictorial space, rather than to the observer’s awareness of their position within the environment. It might be predicted that this variability would be much reduced when depth judgements are made in the real world, and that binocular cues play an important role in providing the observer with information about their point of view relative to the environment. It is also predicted that variability in the apparent point of view will be much reduced in virtual reality than when viewing stereoscopic or non-stereoscopic pictures, and that dynamic binocular cues again play a role in specifying the viewer’s location within the virtual environment.

## Data Availability

Data are available on OSF at osf.io/uyspj.
